# Caffeine and Exercise Performance: Possible Directions for Definitive Findings

**DOI:** 10.3389/fspor.2020.574854

**Published:** 2020-12-11

**Authors:** Gabriel Loureiro Martins, João Paulo Limongi França Guilherme, Luis Henrique Boiko Ferreira, Tácito Pessoa de Souza-Junior, Antonio Herbert Lancha

**Affiliations:** ^1^Laboratory of Applied Nutrition and Metabolism, School of Physical Education and Sport, University of São Paulo, São Paulo, Brazil; ^2^Research Group on Metabolism, Nutrition and Strength Training, Department of Physical Education, Federal University of Parana, Curitiba, Brazil

**Keywords:** caffeine, CYP1A2, ADORA2A, ergogenic substances, sports nutrition, exercise performance, genetic polymorphisms

## Abstract

Caffeine is one of the most studied supplements in the world. Studies correlate its use to increased exercise performance in endurance activities, as well as its possible ergogenic effects for both intermittent and strength activities. Recent findings show that caffeine may increase or decrease exercise performance. These antagonist responses may occur even when using the same dosage and for individuals with the same characteristics, making it challenging to explain caffeine's impact and applicability. This review article provides an analytic look at studies involving the use of caffeine for human physical performance, and addresses factors that could influence the ergogenic effects of caffeine on different proposed activities. These factors subdivide into caffeine effects, daily habits, physiological factors, and genetic factors. Each variable has been focused on by discussions to research related to caffeine. A better understanding and control of these variables should be considered in future research into personalized nutritional strategies.

## Introduction

Active individuals and elite athletes use caffeine purposely to improve performance. Athletes from different modalities consume caffeine, including endurance athletes (e.g., triathletes, cyclists, and marathoners), game athletes (e.g., tennis, volleyball, and handball players), and strength athletes (e.g., weightlifters) (Del Coso et al., 2011). In an evaluation of 20,686 urine samples of elite athletes, 73.8% of the samples contained caffeine in concentrations higher than 0.1 μg·mL^−1^, indicating that three out of four athletes had consumed caffeine before or during sports competition (Del Coso et al., 2011). Moreover, the median urine caffeine concentration increased by ≈21% from 2008 to 2015 (Aguilar-Navarro et al., 2019). Although caffeine's ergogenic effect is well-established, several studies have shown differences in the magnitude of caffeine-mediated effects on exercise performance, where some individuals may not respond, or even negatively respond to the caffeine consumption (Graham and Spriet, 1991; Meyers and Cafarelli, 2005; Wiles et al., 2006; Skinner et al., 2010; Roelands et al., 2011; de Alcantara Santos et al., 2013; Stadheim et al., 2013; Lara et al., 2015).

Studies assessing exercise performance after caffeine/placebo ingestion suggested that ~33% of individuals did not improve their performance (Southward et al., 2018a). This percentage can be questioned and reduced to 5% real lack of response when considering the measurement errors for the performance tests (Grgic, 2018). The possibility of inter-individual variability does not diminish the importance of applying caffeine to performance, but it underlines that caffeine's effects may be unclear under some conditions (Skinner et al., 2010; Roelands et al., 2011).

**Figure 1:**
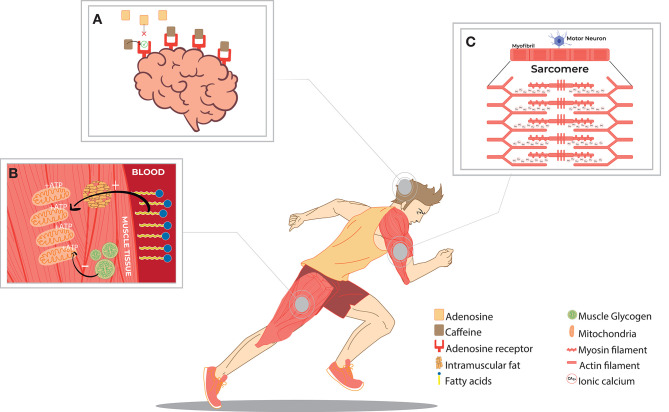
Main mechanisms of action of caffeine-related to increases in training capacities. **(A)** Antagonistic action of caffeine and its secondary metabolites to adenosine at its receptors in the Central Nervous System (CNS), increasing alertness and decreasing perceived exertion in exercise. **(B)** Effect of preserving muscle glycogen from a greater distribution of fatty acids in the bloodstream and energy use under some conditions. **(C)** Increased release of calcium ions by a neuromuscular stimulus, enhancing contraction power in muscle fibers.

There are several hypothetical mechanisms for caffeine-mediated improving performance (Figure 1), including calcium release from the sarcoplasmic reticulum (Klein et al., 1990; von Ruden and Neher, 1993) (Figure 1C), preservation of muscle glycogen through the inhibition of phosphodiesterase (Graham and Spriet, 1991; Cruz et al., 2015) (Figure 1B), and the antagonistic action of caffeine in the adenosine A1 and A2 receptors in the Central Nervous System (CNS) (Daly et al., 1983; McLellan et al., 2016) (Figure 1A). Thus, it is possible that one factor or a combination of these factors may be responsible for the increase in exercise performance after caffeine intake.

In this context, some factors are pointed regarding caffeine's effectiveness as an ergogenic aid, such as dose, training degree, ingestion time, time of day caffeine supply, habitual caffeine consumption, and proposed exercise type (Collomp et al., 1992; Bell and McLellan, 2002; Pataky et al., 2016; Grgic et al., 2019). Additionally, recent findings related to genetic polymorphisms in the *CYP1A2* and *ADORA2A* genes (Womack et al., 2012; Loy et al., 2015) seems to affect the caffeine response to exercise, increasing the need for broader experimental designs, as well as a better understanding of how and for whom caffeine may be appropriate. Therefore, this review aims to discuss the factors that could influence the ergogenic responses to caffeine, developing efficient nutritional strategies with that supplementation.

## Caffeine Effects

### Dosage

The well-accepted dosage of caffeine to improve performance is between 3 and 6 mg.kg^−1^, 60 min before exercise (Goldstein et al., 2010; Maughan et al., 2018). This dosage promotes (between 1 and 8%) performance gains in aerobic exercises, game sports, and exercises with high glycolytic demand (Goldstein et al., 2010; Maughan et al., 2018; Grgic et al., 2019). It is currently established that the benefits of caffeine on performance occur through its direct action on the CNS, improving alertness and reaction time, in addition to reducing the perceived exertion rate (pain) (Maughan et al., 2018).

Reviews have recently highlighted that both low (≤ 3 mg /kg^−1^) and high (i.e., 6–9 mg.kg^−1^) intakes of caffeine dosages can improve exercise performance (Spriet, 2014; Pickering and Kiely, 2018, 2019). From this point of view, Spriet points out that “Smaller doses of caffeine do not alter the responses of the entire peripheral body to exercise” (Spriet, 2014). It reinforces that the effects on improving performance with the consumption of low doses of caffeine are a consequence of its direct action on the CNS.

In this context, Zhang et al. (2020) investigated the effects of low (3 mg.kg^−1^), moderate (6 mg.kg^−1^), and high (9 mg.kg^−1^) doses of caffeine ingestion on cognitive performance and brain activation. Their findings indicate that the ingestion of lower doses of caffeine triggered greater effects on cognition and brain activation when compared to moderate and higher doses (6 or 9 mg.kg^−1^). It suggests that a lower amount of caffeine (≤ 3 mg.kg^−1^) is enough to induce saturation of the effect of caffeine on CNS. It highlights Spriet's positioning (Spriet, 2014), indicating that the use of moderate or higher doses would only be justified through peripheral effects of caffeine on performance tests (see Figures 1B,C). Thus, further investigations involving the administration of higher doses of caffeine (i.e., 6–9 mg.kg^−1^), as opposed to the use of lower doses of caffeine (≤ 3 mg.kg^−1^), should be carried out. Moreover, increasing the caffeine dose should be used based on the individual's tolerance to the substance and the type of physical exercise.

Some studies showed that aerobic exercises with higher doses of caffeine (i.e., 6–9 mg.kg^−1^) could alter substrates' oxidative flow. In those cases, caffeine altered the energetic use of the lipids with the maintenance of the muscle glycogen stocks (Graham and Spriet, 1991; Spriet et al., 1992; Pedersen et al., 2008; Taylor et al., 2011; Cruz et al., 2015). This mechanism triggered improvements in aerobic performance tests when a high dosage of caffeine has been ingested before (Graham and Spriet, 1991; Spriet et al., 1992; Cruz et al., 2015) or after (Pedersen et al., 2008; Taylor et al., 2011) the proposed exercise sessions. In these two cases, both significant variations of the improvement in performance tests with higher doses of caffeine (Graham and Spriet, 1991; Spriet et al., 1992) and the training protocols that consider the maximal lactate steady state among different fitness status (Cruz et al., 2015; San-Millán and Brooks, 2018) should be considered. These studies would allow a definitive position about the effect of caffeine on the oxidation of lipids. This is a very relevant mechanism since it is well-outlined that the drug induces lipolysis, which increases the availability of blood fatty acids, when caffeine is previously, during, or after physical exercise administered (Spriet et al., 1992; Cox et al., 2002; Battram et al., 2004; Gonzalez and Stevenson, 2012; San-Millán and Brooks, 2018). However, favoring energy production from this greater availability of mobilized fatty acids presents conflicting results (Graham et al., 2008; Pedersen et al., 2008; Gonzalez and Stevenson, 2012; Cruz et al., 2015).

Besides, the latest international reports lack a definitive position on caffeine's possible ergogenic effects on strength-to-power exercises (Goldstein et al., 2010; Maughan et al., 2018). This occurs because the current literature is ambiguous regarding the relationship between caffeine intake and improvement in strength-to-power. Whereas some studies (Astorino et al., 2008; Clarke et al., 2015; Trexler et al., 2016; Cesareo et al., 2019) reveal no significant caffeine effects on strength development, others (Anderson et al., 2000; Bruce et al., 2000; Pallarés et al., 2013; Behrens et al., 2015; Cesareo et al., 2019; Wilk et al., 2019) show significant caffeine supplementation improvements. In this context, recent studies have shown that higher doses of caffeine (above 6 mg.kg^−1^) are associated with strength and power performance improvement when compared to the use of moderate doses (Anderson et al., 2000; Pallarés et al., 2013; Durkalec-Michalski et al., 2019; Wilk et al., 2019). In these cases, the use of higher doses of caffeine could be triggering additional performance effects by increasing the calcium release from the sarcoplasmic reticulum (Klein et al., 1990; von Ruden and Neher, 1993). This hypothesis needs to be verified since there is also a recent study (Polito et al., 2019) indicating that the use of lower doses of caffeine (3 mg.kg^−1^) provides same improvements results in muscle strength (chest-press, shoulder-press, and biceps curl exercises) as the administration of moderate doses (6 mg.kg^−1^).

This inconclusive data spawns an interesting situation in which caffeine dosages between 3 and 6 mg.kg^−1^ are not ideal for everyone. Therefore, new studies should take into account that lower doses of caffeine (≤ 3 mg.kg^−1^) act mainly on the CNS (Spriet, 2014) and that the increase in dosage (i.e., 6–9 mg.kg^−1^) may induce peripheral effects (Klein et al., 1990; von Ruden and Neher, 1993; Pedersen et al., 2008; Cruz et al., 2015). Thus, higher caffeine dosage effects should be investigated based on the individual's substance's tolerance and acceptability. Genetic factors (read sections ADORA2A Gene rs5751876 Polymorphism and *CYP1A2* gene rs762551 (g.-163A > C) polymorphism) and daily habits (read section Habitual Caffeine Consumption) must be considered.

### Ingestion Time

The isolated consumption of anhydrous caffeine induces maximum plasma peaks of the substance between 30 and 90 min after consumption of low (2–3 mg.kg^−1^) (Graham and Spriet, 1995; Fletcher and Bishop, 2011), moderate (3–6 mg.kg^−1^) (Graham and Spriet, 1995; Hodgson et al., 2013; Skinner et al., 2013), or high dosages (6–9 mg.kg^−1^) (Graham and Spriet, 1995; Skinner et al., 2013). This caffeine absorption period (represented by the “time necessary to reach the maximum peak of the substance in the plasma”) has been taken into account among the types of exercises in which the ergogenic effect of the substance is already established (endurance and performance in team sports, for example) (Goldstein et al., 2010; Maughan et al., 2018; Southward et al., 2018b; Salinero et al., 2019a). In addition, the use of caffeine occurs mostly 60 min before the performance tests (endurance, team sports, combat sports, and strength-to-power activities) (Grgic et al., 2018; López-González et al., 2018; Southward et al., 2018b; Salinero et al., 2019a). Noteworthy, the current recommendations for optimal caffeine ingestion time in sports performance occur from scientific evidence that investigated the different times of ingestion of the substance from the performance of aerobic performance tests (Bell and McLellan, 2002; Maughan et al., 2018). In this context, Bell and McLellan (2002) reinforce that the caffeine supplementation (5 mg.kg^−1^), 60 min before the cycling exercise, is ergogenic between users and non-habitual users of caffeine. In addition, Bell and McLellan (2002) demonstrated that consumption of the same dosage of caffeine (5 mg.kg^−1^) triggered the same effects on improving performance when the substance was consumed 180 min before the tests compared to its consumption 60 min prior to the tests. Due to our lack of knowledge about the existence of other similar studies (in which different “times of supplementation vs. performance” are investigated), we can speculate that the “ideal” ingestion time for anhydrous caffeine supplementation, at least to start aerobic exercises, is not directly related to the “biggest peaks” of plasma caffeine after its ingestion (30–90 min) (Graham and Spriet, 1995; Bell and McLellan, 2002), but rather to a wider “window” of possibilities, which also involve its metabolism (1–3 h) (Bell and McLellan, 2002). In this respect, it is worth noting that the process of metabolizing caffeine occurs through the reduction of plasma levels of the caffeine, with the gradual increase of its secondary metabolites (Nehlig, 2018). This process apparently occurs with wide variation in the population, due to environmental and/or genetic characteristics (caffeine half-life can vary from 3 to 7 h) (de Mejia and Ramirez-Mares, 2014), being pointed out as an interesting focus for future investigations in studies involving genetics, time of ingestion and sports performance (Pickering, 2019).

In addition to its isolated use, the absorption kinetics of different caffeine doses (1.5–9 mg.kg^−1^) was also investigated combined with carbohydrates (Cox et al., 2002; Desbrow et al., 2009; Skinner et al., 2013). In these cases, the “peak plasma levels” of caffeine have become less pronounced and more elongated, indicating varying times in the absorption of the substance (120–210 min) (Cox et al., 2002; Desbrow et al., 2009; Skinner et al., 2013). The additional use of caffeine compared to the isolated consumption of carbohydrates triggered significant improvements both in the capacity for interval running (Taylor et al., 2011) and in cycling tests (Talanian and Spriet, 2016) between different dosages. In this context, the added caffeine seems to provide benefits, both in muscle recovery between two consecutives training sessions, and in ingestion during cycling tests (Pedersen et al., 2008; Taylor et al., 2011; Talanian and Spriet, 2016). The actual mechanisms behind caffeine use have yet to be investigated since the high dosage of caffeine (8 mg.kg^−1^) has provided increases in muscle glycogen stores (Pedersen et al., 2008), while the use of low dosages (1.5–3 mg.kg^−1^) can improved performance, possibly through the action of caffeine on the CNS (Talanian and Spriet, 2016).

Thus, the “ideal” time for caffeine supplementation aiming at sports performance improvement may depend on the dosage employed and, on the moment (before, during or after the training sessions) (Bell and McLellan, 2002; Taylor et al., 2011; Talanian and Spriet, 2016). In addition, caffeine can also be administered through alternative forms of ingestion, such as food consumption (coffee, teas, and energy drinks, for example), chewing gum, mouth rinses, and aerosol. In some of these cases, there are specific recommendations for intake time before/during exercise. All recommendations are based on the delivery times of caffeine in the bloodstream. For a thorough review, the reader is referred to papers by Wickham and Spriet (2018).

### Withdrawal Effects

Caffeine withdrawal effects are present in all caffeine-related studies. It occurs through caffeine restriction protocols (source foods) in the moments before the performance tests. The problem is that the caffeine restriction period varies in many studies, with depriving source-food of 6 h up to 10-days (Collomp et al., 1992; Bell and McLellan, 2002; Womack et al., 2012; de Alcantara Santos et al., 2013; Pallarés et al., 2013; Cruz et al., 2015; Loy et al., 2015; Pataky et al., 2016). This caffeine deprivation is necessary since its withdrawal in habitual users is related to the increased likelihood of caffeine Withdrawal symptoms, such as episodes of headache, increased sleepiness or tiredness, depression, irritability, and decreased alertness and productivity, nausea, and stiffness (Juliano and Griffiths, 2004; Juliano et al., 2012). These side-effects may impact the performance of control individuals (placebo group), hindering the real understanding of the size of improvement following caffeine administration.

In this context, the lack of studies investigating the effects of caffeine withdrawal on exercise performance is surprisingly critical, especially in order to establish an efficient restriction protocol. The unwanted effects of caffeine withdrawal are generally known to occur more prominently within 12–48 h after the last ingestion (Griffiths and Woodson, 1988; Juliano et al., 2012), with several clinical outcomes, regardless of the habitual caffeine intake (Hughes et al., 1993). In addition, the caffeine half-life can vary from 3 to 7 h depending on some characteristics (de Mejia and Ramirez-Mares, 2014), raising severe doubts about the initial condition of the placebo groups with previous deprivation of caffeine in short periods (6–24 h) (Bell and McLellan, 2002; de Alcantara Santos et al., 2013; Loy et al., 2015; Pataky et al., 2016). This fact seems to be even more relevant, since caffeine and adjacent metabolites play a substantial role in the CNS, “silencing” the adenosine signals while caffeine is present in the body (Daly et al., 1983). However, as most of the reported symptoms are subjective (Juliano and Griffiths, 2004; Juliano et al., 2012) and caffeine blinding is usually difficult, some authors (Dews et al., 2002; Juliano et al., 2019) propose that the effects of caffeine withdrawal could be related to negative expectation effects (nocebo effect).

In this sense, a recent study (Juliano et al., 2019) has successfully investigated the blinding process and caffeine withdrawal effects by evaluating caffeine withdrawal in 87 heavy caffeine consumers (±525 mg.day^−1^), after 16 h from an initial coffee containing 100 mg of caffeine. The research tested the expectation of caffeine withdrawal through the consumption of a cup of coffee containing 280 mg of caffeine or a decaffeinated version of it. Caffeinated and decaffeinated coffees were delivered to the participants and were correctly or wrongly presented to them, creating four possible intake expectations among the individuals: real caffeinated coffee; fake caffeinated coffee; real decaffeinated coffee; fake decaffeinated coffee. For each condition, the participants filled out a standardized questionnaire in accordance with self-reported measurements on the Withdrawal Symptom Questionnaire and Caffeine Craving for 24 h, showing that higher scores were correlated to the expected absence of caffeine consumption (Juliano et al., 2019). This nocebo effect due to the expectation of not consuming caffeine was also addressed in a study that performed the reduction of caffeine in habitual caffeine consumers, and it pointed out greater more withdrawal symptoms among the groups that had the perception of dose reductions during the taper dose (Mills et al., 2019).

This reinforces the need for experimental designs that blind the sample to caffeine administration before performance tests. It is currently unclear whether caffeine withdrawal could cause negatively affect physical performance or whether the reduction of withdrawal symptoms provided by well-blinding the sample could impact exercise performance. This should be focused on future research to avoid serious questions about caffeine's effectiveness on increasing exercise performance (James and Rogers, 2005).

## Daily Habits

### Habitual Caffeine Consumption

Caffeine is a substance presented in a range of *in natura* foods, and industrialized products, commonly consumed by almost every nation in the world (Magkos and Kavouras, 2005; Mitchell et al., 2014). Animal studies indicate chronic caffeine consumption induces neural adaptations correlated to adenosine receptors (Boulenger et al., 1983; Svenningsson et al., 1999). These neural adaptations increase the number of adenosines binding sites, decreasing the development of caffeine stimulating action triggering lower tolerance of caffeine. These neural adaptations increase the number of adenosine binding sites. This may decrease caffeine stimulating action triggering tolerance to its effects. However, there are conflicting results between the studies applied to human performance (Dodd et al., 1991; Bell and McLellan, 2002; Beaumont et al., 2017; Gonçalves et al., 2017; Lara et al., 2019; Grgic and Mikulic, 2020), with some studies showing lesser benefits among regular caffeine users (Bell and McLellan, 2002; Beaumont et al., 2017; Lara et al., 2019) and others suggesting that the habitual consumption does not affect the response to exercise (Dodd et al., 1991; Gonçalves et al., 2017; Grgic and Mikulic, 2020).

In this context, Dodd et al., 1991 were the first to analyze the effects of caffeine tolerance on humans from the recruitment of 17 trained men, eight low-dose consumers (<25 mg.day^−1^), and nine heavy users of caffeine (>300 mg.day^−1^). The individuals were subjected to an incremental cycle ergometer test, with increases of 30 w every 2 min until subjects could not maintain the stipulated cadence. After placebo or caffeine supplementation (3 or 5 mg.kg^−1^) no significant variations in exhaustion time between treatments and placebo group were found, thus characterizing similar responses between usual and non-habitual caffeine users (Dodd et al., 1991). This same indifference was also observed by Gonçalves et al. (2017) from the application of a cycling time-trial (~30 min) performed with 40 male recreational cyclists, 14 low consumers (±58 mg.day^−1^), 12 moderate consumers (±143 mg.day^−1^) and 14 heavy consumers (±351 mg.day^−1^) of caffeine. The study indicated that the caffeine supplementation (6 mg.kg^−1^) showed an improvement in the caffeinated group's average performance compared to both placebo and unsupplemented groups, regardless of the subjects' usual caffeine consumption. Although the study has been well-constructed, the findings related to a possible “myth” regarding caffeine tolerance are still embryonic and therefore cannot be assumed to be valid. Aspects such as the lack of chronic supplementation, a variation of caffeine content in food sources, and the absence of blood circulating caffeine in the participants are strong limitations that must be considered in future studies (McCusker et al., 2003; Areta et al., 2017).

In contrast to the findings above, Bell and McLellan (2002) reported differences between the magnitude of improvement in time to exhaustion at 80% VO_2max_ in an ergometer cycle mediated by caffeine supplementation correlated with habitual caffeine consumption. The study (Bell and McLellan, 2002) consisted of a mixed sample with 21 physically active individuals trained in aerobic activities, where 13 are considered regular caffeine users (≥300 mg.day^−1^) and eight non-users (<50 mg.day^−1^), who performed the physical test 1, 3, or 6 h after the caffeine consumption (5 mg.kg^−1^), placebo or lack of supplementation. A higher ergogenic effect was noticed after receiving caffeine over placebo, with major benefits among non-users and, also, when the exercise-initiated 1 h after caffeine consumption. Beaumont et al. (2017) also evidenced this same phenomenon of tolerance to caffeine habituation in aerobic performance, where 18 active men, low caffeine users (<75 mg.day^−1^), performed a cycle ergometer test at 60% of VO_2peak_ after acute caffeine consumption (3 mg.kg^−1^) or placebo after a 28-day supplementation with caffeine intake (1.5–3.0 mg.kg.day^−1^) or placebo. After the chronic caffeine supplementation period, participants had fewer benefits in the magnitude of their work compared to the test with the same caffeine dosage initially performed (Beaumont et al., 2017). In addition, another recent study (Lara et al., 2019) investigated the chronic administration of caffeine (3 mg.kg^−1^) or placebo during 20 days in both a volitional fatigue cycle ergometer test and at the 15-second Wingate test. The results of the present study showed that chronic caffeine consumption over the stipulated period had an ergogenic effect compared to the placebo condition. But after 4 days of continuous use of caffeine, the ergogenic effects had a lesser extent when compared to the performance tests in initial caffeine supplementation.

In this scenario, chronic caffeine consumption appears to affect improving performance, and it may be necessary to administer acute dosages above those commonly consumed to avoid caffeine tolerance. This seems to be even more important since studies that pointed to the lack of caffeine tolerance used acute dosages above the participants' usual caffeine consumption (Gonçalves et al., 2017; Grgic and Mikulic, 2020). Yet, studies that used the same caffeine dosages (in acute and in chronic administration) indicated less ergogenic caffeine effects after its acute consumption (Beaumont et al., 2017; Lara et al., 2019). Regardless of the usual intake, this hypothesis needs to be tested. In such cases, the time to develop caffeine tolerance can be a dose-dependent way.

### Time of Day Training vs. Caffeine Consumption

Improvement in training-time-dependent physical performance is evidenced in numerous types of exercise. Studies suggest that anaerobic and aerobic activities may enjoy better yields between 16:00 and 20:00 h due to daily variations of the circadian cycle (Racinais et al., 2010; Chtourou and Souissi, 2012; Fernandes et al., 2014). Since caffeine has been identified as a substance capable of affecting circadian rhythm (Narishige et al., 2014) and regulating jet lag (Potter et al., 2016) it is relevant to discuss the caffeine effect related to the period of the day in which that supplement is administrated.

The study of Boyett et al. (2016) observed 20 healthy male subjects [11 trained cyclists (8–407 mg caffeine.day^−1^) and nine untrained cyclists (0–204 mg caffeine.day^−1^)] who performed 3 km time trial test on a cycle ergometer at two different times of day (between 6:00 and 10:00 h or between 16:00 and 20:00 h) under the placebo or caffeine (6 mg.kg^−1^) conditions. The participants demonstrated performance improvement after caffeine consumption compared to placebo, as well as enhanced results in the morning tests compared to the evening tests. In the same year, another study (Pataky et al., 2016) also pointed similar benefits on the 3 km time trial performance test with oral caffeine administration. It demonstrated that circadian factors affect the size of improvement after caffeine intake. Morning caffeine consumption showed improved benefits, compared to the afternoon consumption (Pataky et al., 2016).

In part, caffeine's most significant benefits in the morning may be related to the substance that mitigates performance drops in anaerobic exercises. This is evidenced by an impaired performance during the morning tests in placebo conditions (Chtourou and Souissi, 2012; Fernandes et al., 2014), inducing similar status performances when the caffeine intake occurs in the morning compared to its ingestion in the late afternoon or early evening (Chtourou and Souissi, 2012; Souissi et al., 2013; Fernandes et al., 2014; Mora-Rodríguez et al., 2015). These effects have been documented in the 3 km time trial test (Mora-Rodríguez et al., 2015; Boyett et al., 2016), in strength (Mora-Rodríguez et al., 2012) and in high-intensity short-duration exercises (Souissi et al., 2013, 2019), and less clear gains have occurred during sprints and aerobic exercises (Chtourou and Souissi, 2012; Lopes-Silva et al., 2018).

Besides, it was showed that caffeine consumption in the late afternoon could increase the prevalence of its negative side effects under moderate doses (6 mg kg^−1^) when compared to the same dosages in the morning (increased 13% after caffeine consumption in the morning trials vs. an increase of 26% after caffeine consumption in the afternoon trials) (Mora-Rodríguez et al., 2015). Moreover, the acute consumption of moderate dosages of caffeine (400 mg) up to 6 h before bedtime disturbed sleep compared to placebo groups (Drake et al., 2013). This emphasizes the importance of using caffeine in the morning, aiming at both the better use of performance tests and the maintenance of restful sleep among athletes and active individuals. From this point of view, the existence of possible withdrawal effects (drowsiness) in the night and the application of caffeine in performance tests with sleep-deprived individuals are points of current discussions that should be explored on performance tests and circadian effects (Snel and Lorist, 2011; Crawford et al., 2017).

Through these evidences, caffeine use in the morning can mitigate the unfavorable circadian effects observed in the early phases of the day. Furthermore, it reduces the probability of caffeine to interfere negatively with sleep quality, it is still unclear how caffeine could mitigate the effects of the circadian cycle on sports performance, with the possible role of the substance in cAMP/Ca^2+^ signaling linked to circadian rhythm (Narishige et al., 2014). More research is needed to understand if both the chronic consumption of caffeine and/or the caffeine blood caffeine circulating may affect exercise performance, mainly on anaerobic exercises.

## Physiological Factors

### Degree of Training

Hypothetically, the possible improving performance mediated by caffeine intake may be greater in trained than in untrained individuals, because trained individuals have an improved neuromuscular action potential (Yue and Cole, 1992). On the other hand, trained individuals have a higher concentration of adenosine A2a receptors than untrained ones (Mizuno et al., 2005), which provides evidence that the trained subjects may need more caffeine intake to get the same caffeine effects of untrained subjects. Thus, the mechanisms behind different responses seem to be contradictory.

To make it even more difficult to understand the possible effects of caffeine-mediated increased performance on different training levels, the current academic literature presents contradictory results in the various types of exercise**s** proposed (Collomp et al., 1992; O'Rourke et al., 2008; Astorino et al., 2012; Porterfeld et al., 2013; Brooks et al., 2015; Boyett et al., 2016; Jodra et al., 2020). Some studies suggest greater benefits in trained individuals (Collomp et al., 1992; Astorino et al., 2012) while others propose better performance tests between untrained individuals (Brooks et al., 2015; Boyett et al., 2016). Moreover, there are even studies that showed no effect of differences in performance tests related to fitness level (O'Rourke et al., 2008; Porterfeld et al., 2013; Jodra et al., 2020), indicating that caffeine supply may improve performance tests (O'Rourke et al., 2008; Jodra et al., 2020) or it may even have no effects (Porterfeld et al., 2013) among several fitness levels. It seems to happen regardless of the type of used test, having unclear findings among studies that have investigated both aerobic (O'Rourke et al., 2008; Astorino et al., 2012; Porterfeld et al., 2013) and strength-to-power (Collomp et al., 1992; Brooks et al., 2015; Boyett et al., 2016; Jodra et al., 2020) performance tests. Notably, the recent studies by Jodra et al. (2020) pointed out that elite athletes show more pronounced increases in vigor and vitality when compared to physically active individuals, after ingesting caffeine (6 mg.kg^−1^) in the Wingate test, but this difference was not responsible for better performance on Wingate test. The lack of analysis on several mood dimensions related to different fitness status is a limitation of the studies already done (Collomp et al., 1992; O'Rourke et al., 2008; Porterfeld et al., 2013; Brooks et al., 2015; Boyett et al., 2016).

In addition, the majority of the studies do not report the control of habitual caffeine consumption between different training status groups (Collomp et al., 1992; O'Rourke et al., 2008; Porterfeld et al., 2013; Brooks et al., 2015; Jodra et al., 2020). This lack of control can result in unclear directions about the real influence of training status mediated by caffeine use on performance. In cases of both no controlled and/or unpaired habitual caffeine consumptions, caffeine tolerance itself as well as caffeine withdrawal effects may change the performance tests regardless of the degree of training (see sections Withdrawal Effects and Habitual Caffeine Consumption). Therefore, the influence of fitness status on improving caffeine performance is not clear. Future studies should try to match the amount of caffeine consumed between different fitness statuses and establish caffeine withdrawal protocols before the performance tests. Information on the blinding process of supplementation is also well-regarded in future studies since the placebo effect was suggested by one of the studies among different fitness status (Brooks et al., 2015).

### Gender

The lack of studies involving caffeine in sports performance for women is a topic identified as urgent in future studies (Grgic et al., 2019; Salinero et al., 2019b). For instance, in 2018, only 16.3% of subjects participating in performance-related research were female (Salinero et al., 2019b). One possible explanation is related to the complexity of assessing the effects of caffeine on women since both oral contraceptives (Rietveld et al., 1984; Abernethy and Todd, 1985) and the different phases of the menstrual cycle (Lane et al., 1992; Lebrun, 1993) are designated as factors that could impact caffeine metabolism. In these contexts, the alteration in caffeine's metabolism induces differences in the availability of its secondary metabolites (paraxanthine and theophylline) because the half-life of caffeine in women is greater than in men. Since these factors are justified for different responsiveness to caffeine in different *CYP1A2* polymorphisms (for more details, read section *CYP1A2* Gene rs762551 (g.-163A > C) Polymorphism), it is relevant to take care before extrapolating the male findings to female populations.

From this perspective, new studies should investigate the caffeine effects on women. Currently, caffeine's ergogenic effects in aerobic performance tests do not show a gender bias (Lane et al., 2013; Mielgo-Ayuso et al., 2019; Skinner et al., 2019), even with differences in caffeine half-life related to the use of contraceptives (Skinner et al., 2019). In contrast, caffeine's ergogenic declines in female anaerobic exercise tests, as reported in some research (Sabblah et al., 2015; Chen et al., 2019; Mielgo-Ayuso et al., 2019) but not for all studies (Chen et al., 2015). These contradictory results on anaerobic exercises are based, mainly, in a Systematic Review (Mielgo-Ayuso et al., 2019) that pointed a more pronounced improvement in strength-to-power exercises performance among men, compared to women, considering the same caffeine dosage for both genders. In part, gender differences both in delayed onset muscle pain and in the biomarkers under exercise-induced muscle damage have a greater reduction in male than female athletes (Chen et al., 2019). The lack of randomized, crossover, placebo-controlled studies comparing male to female groups makes it difficult to interpret the real impact of gender on different performance tests. It was also, it was found that caffeine supplementation produces more intense undesirable effects in decreasing the duration/quality of sleep in a gender-dependent way (Kim et al., 2012), which may be related to the decrease in the rate of caffeine metabolism mediated by the use of oral contraceptives (Ali et al., 2015). This needs to be better explored in future studies considering the realization of training at different times of the day, and investigating the impacts of caffeine withdrawal between women and men in performance tests and sleep.

## Genetic Factors

### *ADORA2A* Gene rs5751876 Polymorphism

One of the best accepted theoretical models of caffeine-induced performance improvement is its antagonistic role to adenosine, blocking the adenosine A1 and A2a receptors in the CNS and triggering positive physiological impacts on cognitive ability (Daly et al., 1983; McLellan et al., 2016) and motor activity (Fisone et al., 2004). Thus, it is plausible to assume that polymorphisms in genes encoding adenosine receptors, such as the adenosine A2a receptor (*ADORA2A)* gene, could trigger differentiated reflexes in the metabolic changes induced by the binding of caffeine and its metabolic factors to adenosine receptors, particularly in the CNS.

In this context, Alsene et al. (2003) were the first to point out divergent anxiolytic responses related to different *ADORA2A* genotypes, in a sample of 94 consumers (51 men and 43 women) of low amounts of caffeine (i.e., <300 mg/week). The subjects who had higher anxiety levels after ingesting 150 mg of caffeine were carriers of the T/T genotype, while those with the C/C genotype had lower anxiolytic levels after caffeine supplementation (Alsene et al., 2003). A few years later, Rogers et al. (2010) confirmed the greater caffeine-induced anxiety levels among individuals with the T/T genotype. However, it was not clear whether this increased sensitivity to caffeine among those participants with the T/T genotype was related to the reduced habitual caffeine consumption (Rogers et al., 2010) or due to the genetic trait within the *ADORA2A* gene (Alsene et al., 2003). Because the anxiety stimulus can be interpreted both positively (Cheng et al., 2011) and negatively (Judge et al., 2016; Ngo et al., 2017), the increased caffeine sensitivity among carriers of the T/T genotype should be interpreted with caution. It is proposed that the acute increase in anxiety (pre-exercises or during tests) should be analyzed together with the perceived self-confidence of each individual (Woodman and Hardy, 2003; Kais and Raudsepp, 2004). In this context, the study carried out by Stavrou et al. (2006) points out that self-confidence status may increase or decrease competitive performance and it can be enhanced by anxiety symptoms, being interpreted as “Facilitators” or “Debilitators” for changes in performance. Since it is well-outlined that caffeine can improve the vigor associated with performance (Olson et al., 2010; Duncan and Oxford, 2011; Azevedo et al., 2016), future performance tests should be carried out involving measures of vigor/anxiety for different caffeine dosages and *ADORA2A* gene variants.

Regarding performance tests, Loy et al. (2015) were the first to analyze the relationship between the *ADORA2A* polymorphism and the effects of caffeine supplementation on a 10-min cycling time trial test (Loy et al., 2015). In this study, 12 low-active women using oral contraceptives were supplemented with caffeine (5 mg.kg^−1^) or placebo before the 10-min cycling time trial test, in a randomized, double-blinded and crossover approach. During the test, carriers of the T/T genotype compared to C-allele carriers (i.e., carriers of the C/C or C/T genotype) showed better total work after caffeine supplementation. More recently, these results were not confirmed by Carswell et al. (2020) when assessing 18 physically active men and women who underwent a 15-min cycling time trial, indicating that caffeine supplementation (3 mg.kg^−1^) caused performance improvements regardless of the *ADORA2A* genotype. In addition, recent evidence has suggested that resistance-trained men carriers of the C/C or C/T genotype could respond to caffeine supplementation (3 mg.kg^−1^), but the lack of homozygous individuals for the T/T genotype prevented knowing whether the effect of caffeine is different between the genotypic groups (Grgic et al., 2020a). Currently, we cannot rule out that C-allele carriers can respond differently to caffeine supplementation than carriers of the T/T genotype. Among the characteristics that may differ between studies and influence the results (e.g., sample size, physical fitness level, training protocol, gender differences, use of oral contraceptives or habitual caffeine consumption), a difference that should be noted and considered in future studies is the relationship between the caffeine dosage and the different *ADORA2A* genotypes. For example, the study by Loy et al. (2015) used a moderate dosage of caffeine (5 mg.kg^−1^), while in the study by Carswell et al. (2020) or Grgic et al. (2020a) a reduced dosage was administered (3 mg.kg^−1^).

A single recent study (Banks et al., 2019) correlated caffeine-induced metabolic change to different *ADORA2A* genotypes. The study showed that healthy men carriers of the C/C genotype have less postprandial glucose uptake after consuming caffeine (4 mg.kg^−1^) and carbohydrate (0.75 g CHO.Kg^−1^) when compared to only carbohydrate intake (0.75 g CHO.Kg^−1^). In opposition to the C/C genotype, T-carriers have the same response to glucose uptake with or without caffeine administration (Banks et al., 2019). Even not using performance tests, Banks et al. (2019) indicate that genetic factors related to *ADORA2A* interfere with glucose mobilization after caffeine consumption. This could indicate that genetic backgrounds may influence the contradictory effects of caffeine on muscle glucose metabolism (glucose uptake and increase muscle glycogen stocks) (Graham and Spriet, 1991; Spriet et al., 1992; Gonzalez and Stevenson, 2012). This hypothesis needs to be tested in further studies involving different doses of caffeine, aerobic exercises, and different carriers to *ADORA2A*. For more details regarding the possible peripheral effects of caffeine, see section Dosage.

### *CYP1A2* Gene rs762551 (g.-163A > C) Polymorphism

Following the administration of a single dose, caffeine peak in the bloodstream occurs in ≈60 min, when there is a gradual decrease in blood concentrations and a subsequent increase of its metabolites (Kamimori et al., 2002; Conway et al., 2003; Nehlig, 2018). The main enzymes that convert caffeine to its metabolites are those members of the cytochrome P450 superfamily, mainly the cytochrome P450 family 1 subfamily A member 2 (CYP1A2) (Nehlig, 2018). It was portrayed that a genetic polymorphism in intron 1 of the *CYP1A2* gene (rs762551) could be responsible for a differentiated regulation of the caffeine to its secondary metabolites (i.e., its biotransformation rate) (Daly et al., 1983; Sachse et al., 1999). In these cases, it is pointed out that the secondary metabolites of caffeine (paraxanthine and theophylline) have a higher affinity of binding to adenosine receptors than caffeine itself (Daly et al., 1983). Since the binding of caffeine to adenosine receptors justifies the use of the substance in sports (Figure 1A), individuals with different rates of paraxanthine and theophylline production (via polymorphisms for the *CYP1A2* gene) have been investigated in performance tests. In this regard, it was demonstrated that carriers of the C/C genotype have a lower rate of degradation of caffeine (slow metabolizers) while carriers of the genotype A/A have a rate of accelerated degradation of caffeine (fast metabolizers) (Sachse et al., 1999).

Because of these relevant findings, Womack et al. (2012) investigated whether different *CYP1A2* genotypes could influence caffeine ergogenic response during an endurance exercise. In this study, 35 trained male cyclists (16 A/A homozygote individuals and 19 C-allele carriers based on rs762551) with a low caffeine intake profile (i.e., <150 mg.day^−1^) performed a 40 km morning time trial after 60 min of caffeine (6 mg.kg^−1^) or placebo supplementation. Both *CPY1A2* groups (A/A genotype and C-allele carriers) presented a significant performance increase after caffeine supplementation compared to the placebo condition, but there was a more prominent increase in caffeine-induced performance (i.e., a higher degree of response) among the A/A homozygous participants. This study was the first to show different *CYP1A2* genotypic responses to caffeine supplementation on exercise performance, demonstrating that genetic variability could be, in fact, one of the factors correlated with different caffeine responsiveness. This conclusion was strongly reinforced because all the individuals had the same degree of aerobic training and the same habitual average caffeine consumption (≈86 mg.dia^−1^), regardless of genotype. Both the degree of training and the habitual caffeine consumption were pointed out in previous studies as possible confusing characteristics of caffeine administration on sports performance (see sections Habitual Caffeine Consumption and Degree of Training).

In agreement, Guest et al. (2018) also found different ergogenic effects from endurance exercise caffeine administration based on *CYP1A2* genotypes. In this study (Guest et al., 2018), the participants with the better performance results on a 10 km time trial test after 60 min in three different conditions (placebo, caffeine 2 mg.kg^−1^, or caffeine 4 mg.kg^−1^) indicated that participants with homozygotes for the A-allele had a dose-dependent increase in their performance (PLA = ±17.8 min; 2 mg.kg^−1^ = ±17.0 min; 4 mg.kg^−1^ = ±16.6 min). In addition, between homozygotes for C-allele, there was a dose-response effect related to a drop in performance (PLA = ± 18.3 min; 4 mg.kg^−1^ = ±20.8 min), with no significant effects in individuals with A/C genotypes. This study (Guest et al., 2018) had a great number of participants [101 trained men (A/A = 49, A/C = 44 and, C/C = 8)], with the same standard of caffeine consumption (<100 mg.day^−1^), strengthening previous findings (Womack et al., 2012).

Other studies have analyzed the effects of *CYP1A2* polymorphism in aerobic performance tests with mixed samples (men and women), indicating contradictory results (Algrain et al., 2016; Carswell et al., 2020). Algrain et al. (2016) did not find an improvement in performance with the use of caffeine regardless of genetic characteristics. In opposition Carswell et al. (2020) reinforced improving both in reaction time and in speed of carriers of the A/A genotype (fast metabolizers), compared to the AC or CC genotypes (Slow metabolizers) (Carswell et al., 2020). Aspects such as non-adjustment of dosage by body weight, the absence of caffeine ergogenic response and the administration of caffeine in alternative forms are limitations in the study of Algrain et al. (2016), making it difficult to extrapolate their negative findings. There is currently good evidence (Womack et al., 2012; Guest et al., 2018; Carswell et al., 2020) that aerobic performance tests (with caffeine use) can be influenced by different *CYP1A2* genotypes.

In intermittent activities, Klein et al. (2012) were the first to examine the effectiveness of caffeine supplementation and possible *CYP1A2* genotypic interactions in the performance of trained high school tennis players (8 men and 8 women), all of them with average low caffeine intake (≈97.31 mg.day^−1^). After ingestion of caffeine (6 mg.kg^−1^) or placebo, provided in a randomized, crossover and double-blinded manner participants (7 A/A homozygotes and 9 C-allele carriers) performed morning tests that simulated the training intensity required in a tennis match and caffeine significantly increased the number of successful shots, regardless of genotype (Klein et al., 2012). This finding is in agreement with Puente et al. (2018), where 19 men and women (10 A/A homozygotes and 9 C-allele carriers), caffeine light consumers (<100 mg·day^−1^), were evaluated after ingesting 3 mg·kg^−1^ of caffeine or placebo 60 min before a night training session which consisted of jump tests, direction-change and acceleration tests and a subsequent simulated 20-min basketball game. A similar response between C-allele carriers and A/A homozygote individuals was found (Puente et al., 2018). Until the present moment, there is no evidence of correlation between the *CYP1A2* polymorphism and intermittent exercise performance. However, it should be noted that in both studies there was a low total number of participants (16 and 19 subjects) and the inclusion of different genders in the same analysis. Moreover, there were differences in the exercise protocol, which can hinder a true understanding of the *CYP1A2* genotypic influence on intermittent exercises.

In anaerobic modalities, Giersch et al. (2018) analyzed 20 male subjects (8 A/A homozygotes and 12; C-allele carriers) in two morning 3 km cycling time tests after placebo or caffeine supplementation (6 mg.kg^−1^) and no significant differences was found. Nevertheless, there was a more pronounced increase in serum caffeine among C-allele carriers (±14.17 ppm) than in A/A homozygotes (±11.65 ppm) 1 h after supplementation, without significant differences in metabolites (Giersch et al., 2018). As both training level and habitual caffeine consumption of the groups were similar, it can be speculated that carriers of the C-allele (Slow metabolizers) have differentiated caffeine delivery compared to individuals with the A/A genotype (fast metabolizers), or reduced clearance rate without necessarily affecting the performance of short duration in high-intensity exercises. Another study (Salinero et al., 2017) confirmed the findings above by performing Wingate tests in the afternoon (from 3 to 5 p.m.) and after the ingestion of caffeine (3 mg.kg^−1^) or placebo in 21 male participants (5 A/A homozygotes and 16; C-allele carriers). The results indicate that the caffeine intake increased the peak power (±682 W vs. ±667 W; *p* = 0.008) and the mean power during the Wingate test (±527 W vs. ±518 W; *p* < 0.001) without differences between A/A genotype and C-allele carriers (*p* > 0.05). These appointments were again confirmed by Grgic et al. (2020b) using the same dosage of caffeine (3 mg.kg^−1^) in the morning before the Wingate test. Thus, there is no evidence that different genotyping for *CYP1A2* can mediate increased performance from caffeine use in short duration, high-intensity exercises, at least in the doses used (3–6 mg.kg^−1^). Studies using larger samples and evaluating other doses are welcome before drawing more solid conclusions.

In addition to Wingate tests, Grgic et al. (2020b) also analyzed the impact of caffeine supplementation in the bench press exercise with loads of 25, 50, 75, and 90% of maximum strength (one-repetition maximum), and found no significant differences between *CYP1A2* genotypes—caffeine supplementation improved performance regardless of *CYP1A2* genotypes. As caffeine's effects on strength training still appear to be unclear in the literature (see section Dosage), it is surprising that the increase in muscle performance is documented, especially in the dosages used (3 mg.kg^−1^). As there is a documented increase in strength in a dose-dependent manner with the use of caffeine (Pallarés et al., 2013; Wilk et al., 2019) and the study by Grgic et al. (2020b) reported limitations related to blinding effectiveness, future studies should analyze caffeine effects under different doses and *CYP1A2* genotypes. This becomes even more interesting since Rahimi (2019) demonstrated that supplementation of moderate doses of caffeine (6 mg.kg^−1^) significantly increased strength in individuals with the A/A genotype compared to C–allele carriers.

## Conclusions

**Figure 2:**
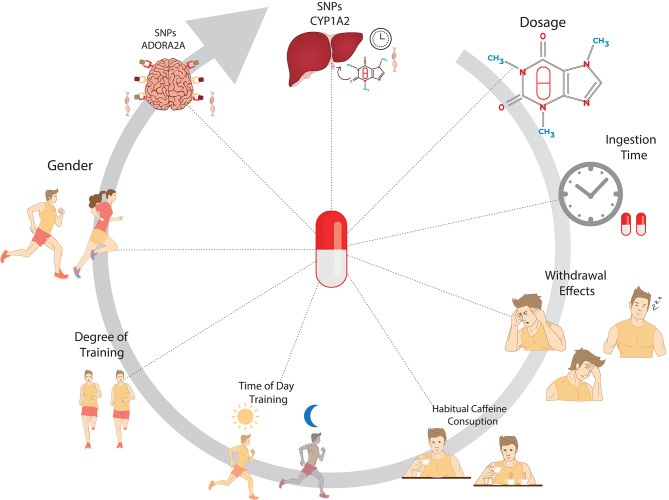
Main factors that could be involved in the ergogenic or ergolitic effects of caffeine supplementation applied to physical exercises. The image represents the variables related to the use of caffeine, such as the applied dosage, ingestion time, and caffeine withdrawal effects. Daily habits such as habitual consumption of food and beverage sources of caffeine and time of training should also be considered. Physiological Factors (gender and degree of training) and genetic factors related to the structures of adenosine receptors in the CNS (ADORA2A) and hepatic enzymes related to caffeine degradation (CYP1A2) are new findings that should be of relevant consideration for the elucidation of inter-individual responses to caffeine on exercise performance.

In conclusion, the ergogenic or ergolitic effects from caffeine use may be influenced by factors related to caffeine effects, daily habits, physiological factors, and genetic factors (Figure 2).

In this context, caffeine's effects enable improvements in exercise performance on a wide dosage range (2–9 mg.kg^−1^). This is curious, since the physiological mechanisms involved in increasing the dosage are not clear. In part, habitual consumption and the time of day when caffeine is ingested may or not diminish the benefits of the substance, however, this does not explain the worsening in the performance observed among some individuals. Possible explanations have been formulated signaling genetic influences related to the *CYP1A2* and *ADORA2A* gene polymorphisms. Currently, there are evidences that the strong influence for the *CYP1A2* gene stands out only in aerobic activities, where T/T genotype would have greater benefits with the increase in the dosage of caffeine, while homozygous individuals for the C-allele would lose their performance with the use of the caffeine. Noteworthy, intermittent and anaerobic exercises seem not to have different responses related to the *CPY1A2* polymorphism. However, the low number of studies and the fact that current approaches have a low number of participants is an important limitation that should be overcome in future studies. Another point related to the lack of results may be the time of caffeine ingestion before conducting short tests (Pickering, 2019). In these cases, it is pointed out that caffeine supplementation is also ergogenic when consumed 1–3 h before exercise (Bell and McLellan, 2002). In addition, physical characteristics (degree of training and gender), caffeine withdrawal, and possible influences related to the *ADORA2A* gene polymorphism present unclear results in the current academic literature. All factors of this review should be considered for experimental research designs aiming at better investigations and/or directions.

## Author Contributions

AL and GM were responsible for the conception of this present work. GM, JG, and AL drafted the manuscript. GM created the images. TS-J, LF, and AL reviewed and made significant contributions to the manuscript. All authors approved the final version of this manuscript.

## Conflict of Interest

The authors declare that the research was conducted in the absence of any commercial or financial relationships that could be construed as a potential conflict of interest.
